# Stroke volume and myocardial contraction fraction in transthyretin amyloidosis cardiomyopathy: A systematic review

**DOI:** 10.3389/fcvm.2023.1085824

**Published:** 2023-01-27

**Authors:** Serenelli Matteo, Cantone Anna, Sanguettoli Federico, Maio Daniele, Fabbri Gioele, Dal Passo Beatrice, Pavasini Rita, Tonet Elisabetta, Passarini Giulia, Rapezzi Claudio, Campo Gianluca

**Affiliations:** Cardiovascular Institute, Azienda Ospedaliero-Universitaria di Ferrara, Ferrara, Italy

**Keywords:** heart failure, amyloidosis, transthyretin, prognosis, stroke volume (SV), myocardial contraction fraction (MCF)

## Abstract

**Background:**

Cardiac amyloidosis (CA) is primarily a restrictive cardiomyopathy in which the impairment of diastolic function is dominant. Despite this, the left ventricular ejection fraction (LVEF) may be depressed in the late stage of the disease, but it poorly predicts prognosis in the earlier phases and does not represent well the pathophysiology of CA. Many echocardiographic parameters resulted important diagnostic and prognostic tools in patients with CA. Stroke volume (SV) and myocardial contraction fraction (MCF) may be obtained both with echocardiography and cardiac magnetic resonance (MRI). They reflect many factors intrinsically related to the pathophysiology of CA and are therefore potentially associated with symptoms and prognosis in CA.

**Objectives:**

To collect and summarize the current evidence on SV and MCF and their clinical and prognostic role in transthyretin (TTR-CA).

**Methods and results:**

We performed a systematic review following the Preferred Reporting Items for Systematic reviews and Meta-Analyses (PRISMA) guidelines. We searched the literature database for studies focusing on SV and MCF in patients with TTR-CA. We analysed the following databases: PUBMED, Cochrane Library, EMBASE, and Web of Science database. Fourteen studies were included in the review. Both SV and MCF have important prognostic implications and are related to mortality. Furthermore, SV is more related to symptoms than LVEF and predicts tolerability of beta-blocker therapy in TTR-CA. Finally, SV showed to be an excellent measure to suggest the presence of TTR-CA in patients with severe aortic stenosis.

**Conclusion:**

Stroke volume and MCF are very informative parameters that should be routinely assessed during the standard echocardiographic examination of all patients with TTR-CA. They carry a prognostic role while being associated with patients’ symptoms.

**Systematic review registration:**

https://doi.org/10.17605/OSF.IO/ME7DS.

## Introduction

Cardiac amyloidosis is caused by the progressive deposition of misfolded proteins, most commonly light chain (AL-CA) or transthyretin (TTR-CA) amyloid. This process disrupts the heart’s structure and function, leading to heart failure (HF), reduced quality of life, and death.

Although been claimed to be a rare disease with an insidious presentation, the availability of new diagnostic tools (i.e., scintigraphy with bone tracer) and the increasing attention to the presence of echocardiographic “red flags” progressively increased the prevalence of the disease during the last decade (1, 2). AL-CA has an estimated annual incidence of 9.7–14.0 cases per million person-years in the United States, and autopsy studies revealed TTR-CA in 25% of subjects over 80–85 years old (3, 4).

The latest guidelines provide a classification of HF still based on LVEF, but this approach does not characterize the pathophysiology of restrictive cardiomyopathies (5). LVEF only describes the change in volumes during the cardiac cycle and is not a precise reflection of the antegrade flow developed during systole. In CA, amyloid deposition in the myocardium causes thickening of the ventricular wall and increased myocardial mass, which results in decreased compliance, diastolic dysfunction and raised filling pressures. Only in the late phases of the disease, with a massive expansion of extracellular volume (ECV), LVEF will decrease. Indeed, the disease progression is accompanied by a progressive impairment of systolic left ventricular function and a decrease in left ventricular diastolic volume, leading to a decline in stroke volume (SV) not necessarily associated with a decreased of LVEF. Recent studies have shown the predictive value of staging system based on biomarkers and several echocardiographic measurements of central cardiac function, but only few studies focused on SV (6–11).

**FIGURE 1:**
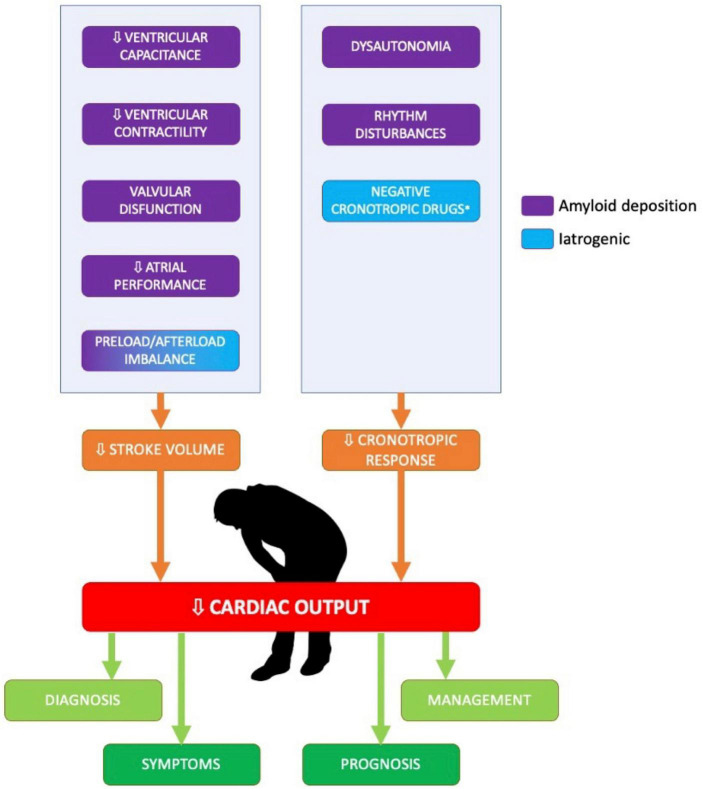
Determinants of low stroke volume and low cardiac output in patients with cardiac amyloidosis. The figure summarized the main determinants of low cardiac output in patients with cardiac amyloidosis.

The SV is a measure of ventricular performance that integrates many factors affecting the ventricle (preload, afterload, contractility, geometry), and that is also representative of the shortening and thickening of the myocardium (Figure 1); indeed, this parameter changes in the earlier stages of the disease (12). Moreover, a newer quantitative SV-derived marker of myocardial function, the myocardial contraction fraction (MCF), has been proposed by King et al. (13); MCF, defined as the ratio between the SV and the myocardial volume (MCF = SV/MV), is a more sophisticated volumetric measure of myocardial shortening which differentiates myocardial performance in similar degrees of hypertrophy.

This report aims to perform a systematic review, analysing the role of the SV and MCF in diagnosing, prognostic stratification, and managing of patients with TTR-CA.

## Methods

We developed a systematic review following the Preferred Reporting Items for Systematic reviews and Meta-Analyses (PRISMA) amendment to the Quality of Reporting of Meta-analyses (QUOROM) statement. The protocol registration application for this study was performed in Open Science Framework (OSF) with the following doi: 10.17605/OSF.IO/ME7D. Two expert cardiologists (M.S., A.C.) independently and systematically searched PUBMED, Cochrane Library, EMBASE, and Web of Science database. The terms searched were: (amyloid*) AND [(transthyretin) OR (TTR)] AND [(echo) OR (stroke) OR (SV) OR (SVi) OR (stroke index) OR (cardiac output)]. The research was carried out in April 2022. Only original articles published in peer-reviewed journals were selected. The shortlisted studies were retrieved as full articles and appraised independently by two unblinded reviewers (A.C. and M.S.), with divergences solved after consensus, according to the following inclusion criteria: (i) English language; (ii) reporting data on echocardiographic-derived or CMR-derived SV/SVi and/or MCF; (iii) involving patients with TTR-related cardiac amyloidosis (wtTTR-CA, vTTR-CA), (iv) data published in peer-reviewed journal. SV is defined as the volume of blood pumped out of the left ventricle during each systolic cardiac contraction. It can be calculated by a doppler-derived method (representing specifically the antegrade SV) or as the difference between end-diastolic volume (EDV) and end-systolic volume (ESV). MCF is defined as the ratio of SV to myocardial volume (MV). Myocardial volume is generally calculated as the LV mass divided by the mean density of the myocardium (1.04 g/ml).

Exclusion criteria for this study were: (i) duplicate reports, (ii) gray literature; (iii) only abstract or posters; (iv) review or case report/series; (v) editorials. Outcomes of interest were diagnostic, prognostic and clinically meaningful findings correlated to SV/SVi and MCF in patients with TTR-CA. In particular, the aim of this systematic review is to describe available evidence relating SV/SVi and MCF to (i) symptoms, (ii) differential diagnosis and (iii) prognosis.

## Results

### Results of the search strategy

**FIGURE 2:**
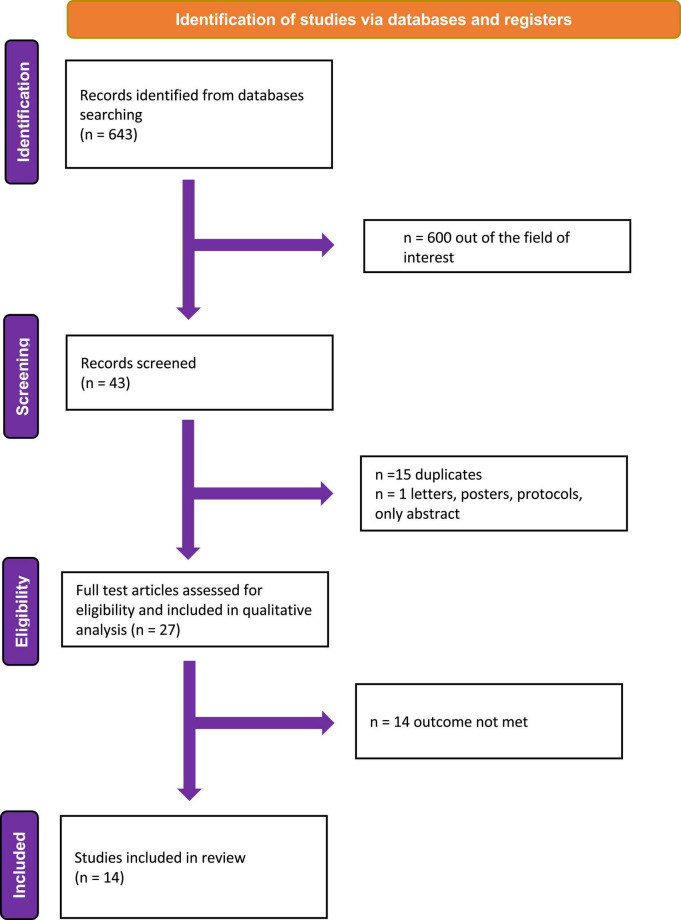
Study flow diagram.

Overall, 643 citations were obtained. After the first screening, 600 records were excluded because they were out of the field of interest; the remaining 43 records were further examined. Of these, 16 were excluded with reasons (15 = duplicates, 1 = only abstract). Finally, of the 27 studies examined as full-text, 13 were excluded because they did not report any outcome of interest (Figure 2). Fourteen studies were finally included in the review (12, 14–26). Three studies provided data on the relation between symptom and SV (18, 19, 26). Three focused on the prognostic role of SV (14, 15, 20) and three on the prognostic role of MCF (12, 16, 17, 21). Only one study addressed the implication of the use of neuro-hormonal antagonists (i.e., beta-blockers) in CA patients, according to SV (24). Two study showed the diagnostic usefulness of SV in patients with aortic stenosis (AS) and CA (22, 23).

One compared right heart catheterization-derived (RHC) SV with doppler-derived SV in patients with CA (25).

Five studies out of 11 used the pulsed wave doppler measurement of LVOT velocities and LVOT diameter measurements (20, 23, 25, 26). Six studies calculated SV by linear left ventricular (LV) dimension measured by M-mode. One study used a bioimpedentiometry technique (18). One study used a doppler-derived SV for MCF calculation while six used SV calculated as difference between EDV and ESV (23). Table 1 summarizes the main findings of each study and the methods applied for SV and MCF calculation.

**TABLE 1:** Summary results of the selected studies.

References	Parameters	*N* ^°^	Summary of the study finding	Overview of univariate/multivariate regression
Castano et al. (22)	SV*, MCF*	151 -84% no CA -16% TTR-CA	Significant univariate predictors of ATTR-CA included SVI <35 ml/m^2^ and a decreased MCF, but in multivariable logistic regression only average mitral annular S’ remained significantly associated with ATTR-CA.	**Predictors of ATTR-CA (univariate analysis).** SVi < 35 ml/m^2^: OR 4.53, 95% CI 1.68–12.21; *P* = 0.003. MCF: OR for 1% unit decrease 1.10, 95% CI 1.05–1.15; *P* < 0.001.
Tendler et al. (16)	MCF*	66 -27% wtTTR-CA -21% vTTR-CA -52% AL-CA	There was no significant difference in LVEF between patients who survived the study period and those who died, while there was a significant difference in MCF. At the univariate analysis MCF, as a continuous parameter, was significantly associated with death while LVEF was not, and at the multivariate analysis, an MCF <30 was an independent risk predictor of death, driven by a higher risk in AL-CA subjects than ATTR-CA.	**Predictors of death (univariate analysis).** MCF: HR for each 1% increase 0.972, 95% CI 0.947–0.998; *P* = 0.035. **Predictors of death (multivariate analysis).** MCF < 30%: HR 2.841, 95% CI 1.214–6.648; *P* = 0.016.
Aimo et al. (24)	SV*	99 -64% wtTTR-CA -3% vTTR-CA -33% AL-CA	AL amyloidosis, reduced function of left heart (lower SV and FE) and right heart function (TAPSE) were predictors of adverse events during beta-blocker therapy and were associated with HF hospitalization; lower systolic blood pressure predicted need for dose reduction.	**Predictors of cardiovascular events or need for dose reduction** **during BB therapy (univariate analysis).** SV: negatively associated with events (*P* = 0.036). **Predictors of HF hospitalization in patients started on BB** **(univariate analysis).** SV: negatively associated with events (*P* = 0.027). CO: negatively associated with events (*P* = 0.017).
Monfort et al. (18)	SV	33 -45% no CA -55% vTTR-CA	At CPET, ATTRv-CA patients had reduced changes (relative to increase in VO^2^) in CI and SV compared with controls (suggesting a poor inotropic myocardial reserve).	**Peak SV index,% change from rest.** vTTR-CA vs. Controls: 18.0% ± 6.5 vs. 30.5% ± 5.9 *P* < 0.0001. **Peak cardiac index,% change from rest.** vTTR-CA vs. Controls: 92% ± 31 vs. 144% ± 22; *P* < 0.0001.
Ruberg et al. (20)	SV	29 -62% wtTTR-CA -38% vTTR-CA	Statistically significant univariate predictors of mortality for the entire cohort at baseline were disease duration, HR > 70, baseline SV, LVEF < 50%, presence of V122L mutation.	**Predictor of death (univariate analysis).** SV: HR for 1 ml increase 0.96, 95% CI 0.92–1.00; *P* = 0.05.
Bhuiyan et al. (14)	SV*	29 -62% wtTTR-CA -38% vTTR-CA	At multivariable survival analysis, baseline LVEF <50% was associated with increased mortality. Declines in LVEF were lower than decrease in SV; declines in LVEF were strongly correlated with declines in SV, but not with declines in end-diastolic volume.	**Correlation analysis.** SV correlation with LVEF: r = 0.769, *P* = 0.0093. EDV correlation with LVEF: r = –0.306, *P* = 0.389.
Siepen et al. (21)	SV*, MCF*	191 -100% wtTTR-CA	LVEF, SVi, and MCF weren’t predictors of mortality.	**Predictor of death (univariate analysis).** SVi (ml/m^2^) c-statistics = 0.429. MCF (%) c-statistics = 0.383.
Arenja et al. (19)	MCF*	330 -30% control -24% wtTTR-CA -8% vTTR-CA -24% AL-CA -18% HCM -12% IHD	In HF, MCF discriminates CA from other forms of LVH (better than LVEF) and comparable to LVMI in discriminating LVH from controls. Cut-off value for MCF <50% and for LVEF <60% could best identify patients with a high probability for CA.	**Discrimination between HCM and CA.** Diagnostic performance of MCF for discriminating AL from HCM: AUC 0.84, *P* < 0.0001).
Rubin et al. (17)	MCF*	530 -30% wtTTR-CA -70% vTTR-CA	Most of the patients who died during follow-up had a lower value of MCF and a lower mean MCF at baseline compared to survivors. LVEF was lower at baseline in those who died, but still in the normal range in both cohorts. At univariate analysis, MCF <25% had a greater predictive value for mortality than EF <50%. At multivariate analysis MCF <25% was independently associated with a greater risk of death.	**Predictor of death (univariate analysis).** MCF < 25%: HR 8.46, 95% CI 4.8–14.9, *P* < 0.0001. **Predictor of death (multivariate analysis).** MCF < 25%: HR 5.37, 95% CI 1.82–15.9, *P* = 0.0024.
Nitsche et al. (23)	SV, MCF	191 -8% wtTTR-CA -1% AL-CA -91% no CA	The usefulness of SVi for the detection of CA-AS was tested; while GLS did not reliably differentiate AS from CA-AS, SVi showed good discriminative power by ROC analysis (0.77, 95% CI 0.69–0.86; *P* < 0.002), comparable to extracellular volume by CMR. SVi was also associated with CA at univariate logistic regression analysis and at multivariate analysis.	**Prediction of cardiac amyloidosis (univariate analysis).** OR for SVi increase 0.21, 95% CI 0.08–0.56; *P* = 0.002. **Prediction of cardiac amyloidosis (multivariate analysis).** OR for SVi increase 0.30, 95% CI 0.10–0.87; *P* = 0.027.
Clemmensen et al. (26)	SV	44 -23 wtTTR-CA -11% vTTR-CA -20% AL-CA -45% no CA	CA patients had reduced CI (*P* < 0.01) as a result of severely reduced SVi. They also presented lower VO^2^ than controls (15 ± 6 vs. 33 ± 7 mL/min/kg bwt; *P* < 0.0001). Furthermore, CA patients had a severely reduced inotropic myocardial reserve. Only small exercise-induced increases in left ventricular stroke work index (LVSWI) and preload-adjusted left ventricular stroke work (LV-PASW) were seen in CA patients. The poor LVSWI and LV-PASW reserve was mainly attributable to only a small increase in SV during exercise.	**Increase in SVi during exercise (controls vs CA patients).** ΔSVi: 4 mL/m^2^ (range: –1 to 8) vs. 14 mL/m^2^ (range: 5– 25); *P* < 0.0001. **Increase in CI during exercise (controls vs CA patients).** ΔCI: 2 ± 2 vs. 7 ± 2 L/min; *P* < 0.0001.
Granstam et al. (25)	SV	14 -36% wtTTR-CA -64% AL-CA	Assessment of echocardiographic-derived SV is feasible and comparable to RHC-derived SV in patients with CA.	**SVi estimation comparison (echo Doppler-derived vs right** **heart catheterization).** SV was similarly slightly reduced in both catheterization (66 mL, IQR 51–89) and echocardiographic assessment (65 mL, IQR 25–125).
Chacko et al. (15)	Svi*, MCF*	1,240 -62% wtTTR-CA -38% vTTR-CA	SVi, right atrial area index, LS and severe AS were independently associated with patient survival in the overall population; E/e’ was associated with survival if severe AS patients were excluded. LS, SVi, and severe AS remained independently associated with survival also after adjustment for NYHA class and for NAC staging system (eGFR and NT-proBNP).	**Risk of death (univariate analysis).** SV: HR for 1 ml increase 0.95 (95% CI 0.93–0.96).
Knight et al. (12)	SV*, MCF*	322 -35% wtTTR-CA -23% vTTR-CA -41% AL-CA	At the univariable analysis, SVi and MCF were predictive of mortality. At multivariable Cox model analysis adjusted for age and sex, SVi remained independently predictive of mortality while in a multivariable model, the only parameter that remains independently predictive of mortality was TAPSE.	**Risk of death (univariate analysis).** SVi: HR for 5 ml decrement 1.40, 95% CI 1.24–1.57; *P* < 0.001. MCF: HR for 10% decrement 1.55, 95% CI 1.32–1.81. **Risk of death (multivariate analysis).** SVi: HR for each 5 ml/m^2^ decrement 1.24; 95% CI 1.04–1.48, *P* = 0.019. MCF: HR for each 10% decrement 1.25; 95% CI 1.00–1.57, *P* = 0.053.

SV* and MCF*, in these studies the estimation of stroke volume and MCF was from linear left ventricular (LV) dimensions measured by M-mode echocardiography; AL-CA, light chain cardiac amyloidosis, AS, aortic stenosis; ATTR-CA, transtiretin cardiac amyloidosis; CI, cardiac index; CO, cardiac output; CPET, cardio-pulmonary exercise test; EDV, end-diastolic volume; eGFR, estimated glomerular filtration rate; ESV, end-systolic volume; HCM, hypertrophic cardiomyopathy; HR, heart rate; IHD, ischemic heart disease; LS, longitudinal strain; LV-PASW, left ventricular pressure-adjusted stroke work; LVEF, left ventricular ejection fraction; LVOT, left ventricular outflow tract; LVSWI, left ventricular stroke work index; MCF, myocardial contraction fraction; MV, myocardial volume; NT-proBNP, N-terminal pro-brain natriuretic peptide; RHC, right heart catheterization; SV, stroke volume; TAPSE, transannular plane excursion; vTTR, variant TTR; wtTTR, wild type TTR.

## Discussion

### Stroke volume and myocardial contraction fraction assessment

Although there are no specific guidelines for SV assessment in patients with HF, echocardiographic recommendation for aortic stenosis grading suggest to deriving SV by the pulsed-wave doppler measurement of LVOT velocities and LVOT diameter measurements (27). This method was applied in 5 out of 11 studies (20, 23, 25, 26), one study used a bioimpedentiometry technique (18), and the remaining six studies calculated SV by the linear left ventricular (LV) dimensions measured by M-mode echocardiography (12, 14, 15, 21, 22, 24).

While the doppler-derived estimates are more representative of the real antegrade flow through the aortic valve during the cardiac cycle, others estimate based solely on the difference between EDV and ESV are representative of both antegrade and retrograde SV, and therefore are more influenced by the presence of significant mitral regurgitation. Notably, Granstam et al. (25) found RHC SV to be comparable to doppler-derived SV assessment in patients with CA: cardiac output (CO) and cardiac index (CI) assessed by RHC were both reduced in patients with amyloidosis [4.3 (3.3–6.7) L/min and 2.2 (1.0–3.8) L/min/m^2^], and the calculated flows comparable to those obtained with echocardiography. At the same time, SV was similarly slightly reduced in both catheterization and echocardiography estimates [66 (51–89) and 65 (25–125) mL, respectively].

Interestingly, only one study out of seven examining MCF, used a doppler-derived SV for its calculation (12). The remaining six studies computed SV as the difference between EDV and ESV.

#### Stroke volume and symptoms

**FIGURE 3:**
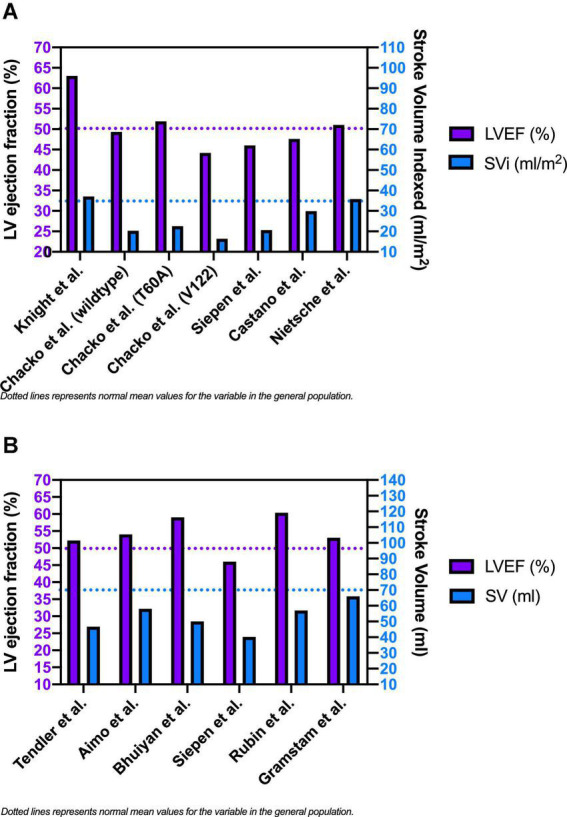
Baseline left ventricular ejection fraction, stroke volume index [panel **(A)**] and stroke volume [panel **(B)**] in patients with cardiac amyloidosis.

Figure 3 shows the mean baseline SVi (or SV if SVi was not reported) and LVEF values extrapolated from the selected studies population. While mean LVEF was generally preserved or slightly reduced, mean SV was significantly lower than typical reference values in most of the study populations. Starting from the assumption that the baseline low SV and its reduced reserve during exercise are among the main determinants of the reduced exercise tolerance of patients with CA, Clemmensen et al. (26) tried to evaluate the link between impaired exercise capacity and hemodynamic alterations during functional stress in patients with CA. Patients with CA usually develop symptoms with physical activity because of rising filling pressures, which are necessary to maintain adequate SV. The authors studied 44 subjects, 24 with confirmed CA and 20 without CA (control group). The first group comprised wtTTR-CA (*n* = 10), vATTR-CA (*n* = 5) and AL-CA (*n* = 9) patients. CA patients had reduced CI (*P* < 0.01) due to severely reduced SVi. They also presented lower VO^2^ max (normalized per body weight) than controls (15 ± 6 vs. 33 ± 7 mL/min/kg; *P* < 0.001) and had a severely reduced inotropic myocardial reserve.

Starting from the previous finding of a lower rate of oxygen consumption at peak exercise (peak VO^2^) in wtTTR-CA, vTTR-CA, and AL-CA, Monfort et al. (18) performed exercise testing with oxygen consumption measurement and SV measurement by bioimpedentiometry in African-American patients with vATTR-CA. At peak exercise, CI increased by approximately 2-fold compared to a 3-fold increase in age-matched controls. All patients were not receiving beta-blocking therapy, and the two groups reached similar peak HR. Furthermore, HR recovery expressed as a percent decrease in peak HR at 1 and 3 min post-exercise was blunted in vATTR-CA patients compared with the control group.

Finally, in the study by Arenja et al. (19), a significant correlation was found between MCF and NYHA class, and therefore with symptoms. MCF was significantly reduced according to the increase in NYHA class in a cohort of CA patients (19). The same correlation was not present between LVEF and NYHA classes.

To summarize these findings, patients with amyloidosis presents with low SV and low CI despite usually normal LVEF. They also have low SV and CI reserve. These parameters are linked to reduce peak exercise VO^2^ and exercise tolerance. Consistently, also the MCF is significantly reduced according to the increase in NYHA class.

#### Prognostic stratification

##### Stroke volume

Recent studies have shown the predictive value of several echocardiographic features, such as LVEF, average strain rate, E/e’, TAPSE, and SVi (10, 11). In 2011, Bhuiyan et al. (14) wanted to evaluate the end-diastolic pressure-volume relation and other pressure-volume indices in patients with TTR-CA to determine how these indices change over time and whether abnormal pressure-volume relations and indices of pump function were associated with reduced survival. They studied 29 patients with TTR-CA (both wtTTR-CA and vTTR-CA forms) over 18 months, and found that, at multivariable survival analysis, initial LVEF <50% was associated with increased mortality (HR 6.6, 95% CI 1.1–40.3). They also found that declines in LVEF were of a lower magnitude than the decrease in SV because of concomitant reductions in EDV over time. In fact, declines in LVEF were strongly correlated with declines in SV (r = 0.769, *P* = 0.0093), but not with declines in EDV (r = –0.306, *P* = 0.389). This shows that SVi changes in the earlier stages disease, and it might be an early predictor of a decrement in LVEF and, consequently, of the patient’s outcome.

One year later, Ruberg et al. (20) tried to find clinical, echocardiographic, or biochemical baseline parameters that could predict the course of the disease, examining 29 patients with TTR-CA (11 vTTR-CA and 18 wtTTR-CA). They showed for the first time that SV could be a useful tool for the prognostic stratification of CA. Indeed, they found SV to be a predictor of death at univariate analysis (HR 0.96 for each ml increase, 95% CI 0.92–1.00, *P*-value 0.05). In 2020, Chacko et al. (15) studied a larger sample of cases with more than 1,000 patients with TTR-CA from the National Amyloidosis Center (NAC) of London (62% of patients had wtATTR-CA, 25% had V122l-associated vTTR-CA, 10% had T60A-associated vTTR-CA, and 3% had non-V122I non-T60A-associated vATTR-CA). In this study SVi, right atrial area index, longitudinal strain and severe aortic stenosis (AS) were independently associated with patient survival in the overall population after adjustment for NYHA class and a validated staging system (including eGFR and NT-proBNP), highlighting their independent prognostic role for survival prediction. Interestingly, this study also showed different degrees of disease severity across the different genotypes: compared to wtTTR-CA, patients with V122I mutation had similar increases in LV wall thickness but significantly lower indices of LV function (including SVi, LVEF, and MCF). Notably, in V122I patients, SVi resulted as a weaker predictor of mortality than wtTTR-CA. One possible explanation could be that in the study, SVi was calculated as the difference of VTD and VTS indexed to BSA and not with a PW-doppler approach; therefore, a possible overestimation of SVi could have happened in those with moderate-to-severe mitral regurgitation, negatively affecting the predictive value of this parameter in vTTR-CA. Significant MR was indeed more frequent in patients V122I vTTR-CA versus wtTTR-CA patients (40.7 vs. 27.1%).

Interestingly, following this publication from the NAC of London, Rosenblum et al. (28) performed an analysis of the echocardiographic-derived pressure-volume loops of the same cohort. All patients with ATTR-CA demonstrated impaired diastolic properties with leftward shifted end-diastolic pressure relationship (EDPVR), especially for those with V122I variant, which presented the lowest chamber function and stroke volume (28).

##### Myocardial contraction fraction

Myocardial contraction fraction might give important prognostic information for CA patients. The progressive amyloid deposition in the myocardium causes an increase in left ventricular MV and a decline in SV with a deterioration of the ventricular function and, therefore, a decline in MCF (17). The first to study the predictive power of MCF in cardiac amyloidosis were Tendler et al. (16) in 2014. They studied a small population of 66 patients with AL-CA and TTR-CA, hypothesizing that MCF would be superior to LVEF in predicting survival among patients with CA. Interestingly they did not find a significant difference in LVEF between patients who survived the study period and those who died, while they found a significant difference in MCF. At the univariate analysis, MCF, as a continuous parameter, was significantly associated with death while LVEF was not, and at the multivariate analysis, an MCF <30% was an independent risk predictor of mortality, driven by a higher risk in AL subjects than ATTR amyloidosis. MCF did not differ between patients with AL and ATTR amyloid, even though subjects with TTR-CA had a larger increase in MV than subjects with AL, corroborating the hypothesis of a direct detrimental effect of light chains on myocardial function. The direct effects of light chains on cardiac performance have already been demonstrated before, but this data highlights the MCF power to measure myocardial contractility and the consequences of amyloid infiltration on myocardial performance, regardless of the different mechanisms by which this occurs. After this study, the interest in MCF grew, as it seemed to be a revolutionary parameter capable of prognostically stratifying the patient with CA more subtly and completely. It was, therefore, unexpected when Siepen et al. (21), in 2017, published their study with the intent to analyse clinical predictors of mortality in 191 patients with TTR-CA and showed that both SV and MCF were not significantly correlated with survival. It is essential to notice that Siepen’s study population was bigger than the Tendler’s, but with a limited number of fatal events and little statistical power. Furthermore, this study did not use the doppler-derived method for SV calculation.

Two other studies analysing this parameter were published in less than a year to clarify its role. Knight et al. (12) studied 322 patients and analysed 11 commonly measured (at CMR and echocardiography) structural and functional cardiac parameters, which were categorized into three groups, according to their likelihood of being abnormal across the degree of myocardial infiltration (low burden/intermediate/high burden variables) (12). Cardiac amyloidosis burden was quantified using CMR-derived extracellular volume. In the univariate analysis, the SVi, and MCF were predictive of mortality. In multivariate regression SVi was an independent predictor of mortality (HR for each 5 ml/m^2^ decrement 1.24; 95% CI 1.04–1.48, *P* = 0.019), and in the model including MCF, this last one did not reach statistical significance for a few points (HR for each 10% decrement 1.25; 95% CI 1.00–1.57, *P* = 0.053).

Finally, Rubin et al. (17) published a study with the same Tendler’s hypothesis (that MCF could be a better predictor of survival than LVEF) but with a larger population counting 530 patients, all presenting TTR-CA. They found that most of the patients who died during follow-up had a lower value of MCF and a lower mean MCF at baseline versus those who did not. The LVEF was lower at baseline in those who died but still in the normal range in both cohorts. In multivariate analysis, MCF <25% was independently associated with a greater risk of death. Therefore, the prognostic role of this parameter seems to have been confirmed. Still, it is crucial to notice that, in all these studies, MCF has been calculated using LV mass and volumes not directly measured and consequently subjected to error. It is undoubtedly attractive that MCF, even if measured with the simplest method, can predict adverse outcomes, but studies analysing actual volumetric chamber data are lacking.

### Stroke volume in patients treated with beta-blockers

Except for tafamidis, which is currently the only disease modifying treatment available for cardiac amyloidosis, most of the medical management of CA patients is based on treatment of its complications (e.g., hemodynamic deterioration, arrhythmias, and systemic embolism). On this regard, the systematic use of neurohormonal antagonist in the setting of CA is still debated. Specifically, beta-blockers are perceived to be poorly tolerated or contraindicated in the setting of CA because of the fear of hypotension, conduction disturbances or impossibility of adequately increasing CO, especially because of the typical restrictive pathophysiology observed in these patients. In the observational study of Aimo et al. (24) patients started on a beta-blocker (56%) did not show a higher frequency of hypotension (*p* = 0.97), fatigue (*p* = 0.83), syncope (*p* = 0.13), symptomatic bradycardia (*p* = 0.65), need for pacemaker implantation (*p* = 0.51), or HF hospitalization (*p* = 0.59) compared to the others. On the other hand, in this study, SV (*p* = 0.027) ad CO (*p* = 0.017) resulted predictors of HF, while CO was predictive of syncope in patients treated with beta-blockers (24). These findings show that in CA patients treated with beta-blockers, SV and especially CO are related to symptoms, and the use of rate-limiting drugs should be carefully evaluated on a tailored base.

#### Diagnostic role of SV and MCF in patients with coexistent AS or unexplained LV hypertrophy

Stroke index and MCF might be useful tools to raise the diagnostic suspicion of CA also in patients with hypertrophy. It is estimated that almost 15% of the AS population and 30% of the subset with “low-flow low-gradient” pattern may have CA (29). In these patients, significant myocardial thickening is naturally attributed to long-standing pressure overload and is recognized as a potential sign of a storage disease. Coexisting CA and AS has been associated with worse outcomes (22, 23). Castano et al. (22) used 99mTc-PYP scintigraphy to examine 151 elderly patients with severe symptomatic AS undergoing TAVR, and they found a prevalence of TTR-CA of 16%, and a greater percentage of this group had low-flow low-gradient AS. In this study, Castano proposed an evaluation model consisting of echocardiographic parameters comprising s’, SVi, and MCF to select patients with TTR-CA and, consequently, refer for a 99mTc-PYP amyloid scan before TAVR. Using logistic regression models, significant univariate predictors of TTR-CA included a SVi <35 ml/m^2^ (OR 4.53, 95% CI 1.68–12.21; *P* = 0.003) and a decreased MCF (OR for 1% unit decrease 1.10, 95% CI 1.05–1.15; *P* < 0.001). Nitsche et al. studied 191 consecutive patients with AS scheduled for TAVR. The 81.7% of this population underwent complete standardized assessment (echocardiography, ECG, CMR, 99mTc-PYP, serum and urine free light chain measurement, biopsy in AL) (23). The authors tested SVi for the detection of CA. While longitudinal strain did not reliably differentiate AS from CA-AS, SVi showed good discriminative power by ROC analysis, comparable to extracellular volume by CMR. SVi was also associated with CA by univariate logistic regression analysis (OR 0.21, 95% CI 0.08–0.56; *P* = 0.002) and by multivariate analysis (OR 0.30, 95% CI 0.10–0.87; *P* = 0.027).

In 2017, Arenja et al. (19) studied with CMR 230 patients with left ventricular hypertrophy (LVH), including 132 patients with a confirmed diagnosis of CA [AL-CA (*n* = 80), vTTR-CA (*n* = 27), wtTTR-CA (*n* = 25)], 60 with hypertrophic cardiomyopathy and 38 with hypertensive heart disease (HHD). The mean value of MCF was reduced in all groups (HCM, 80.0 ± 20.3%; TTR-CA, 74.9 ± 32.2%; HHD 92.6 ± 20%; with *P* < 0.05 for all), and the lowest MCF value was in patients with AL-CA (50.5 ± 20%, *P* < 0.05 vs. all other groups).

Myocardial contraction fraction outperformed LVEF and left ventricular mass index (LVMI) in discriminating between different etiologies of LVH and between AL-CA and other forms of LVH (AUC = 0.84, *P* < 0.001). Moreover, cut-off values for MCF < 50% and LVEF < 60% allowed for identifying patients with a high probability of CA. This higher ability of MCF to discriminate AL-CA from other forms of LVH can be explained by a higher grade of LV geometric deformation or a greater level of contractility dysfunction in AL-CA, with an increase in LV mass and a decrease in end-diastolic LV volume that appears more pronounced than in other forms of LVH.

## Conclusion

The findings of this systematic review highlight the role of SV and MCF in the diagnosis and prognostic stratification of patients with CA. Being the results of the several factors, SV and MCF should be considered very informative parameters to be routinely assessed during a standard echocardiographic examination of all patients with TTR-CA. They carry both a diagnostic and a prognostic role while being associated with patients’ symptoms. With the advance and availability of disease-modifying treatment for TTR-CA, they may also emerge as possible parameters to evaluate disease progression and response to treatments. This should be confirmed in further exploratory studies. It is essential to notice that discrepancies between some trials may be partly explained by the different methods used to estimate SV, which was not performed by a doppler-derived technique in most studies. Finally, data correlating SV and MCF with heart failure hospitalization are lacking and should be investigated further.

## Data availability statement

The original contributions presented in this study are included in this article/supplementary material, further inquiries can be directed to the corresponding author.

## Author contributions

SM and CA conceived and design, systematic literature research, and critical writing and revising the intellectual content. SF, MD, FG, DB, PR, TE, PG, and CG revised the intellectual content. RC conceived and design. All authors contributed to the article and final approval of the version to be published.
